# Pro-inflammatory cytokines derived from West Nile virus (WNV)-infected SK-N-SH cells mediate neuroinflammatory markers and neuronal death

**DOI:** 10.1186/1742-2094-7-73

**Published:** 2010-10-31

**Authors:** Mukesh Kumar, Saguna Verma, Vivek R Nerurkar

**Affiliations:** 1 Retrovirology Research Laboratory, Department of Tropical Medicine, Medical Microbiology and Pharmacology, John A. Burns School of Medicine, University of Hawaii at Manoa, 651 Ilalo Street, BSB 325AA, Honolulu, Hawaii 96813, USA

## Abstract

**Background:**

WNV-associated encephalitis (WNVE) is characterized by increased production of pro-inflammatory mediators, glial cells activation and eventual loss of neurons. WNV infection of neurons is rapidly progressive and destructive whereas infection of non-neuronal brain cells is limited. However, the role of neurons and pathological consequences of pro-inflammatory cytokines released as a result of WNV infection is unclear. Therefore, the objective of this study was to examine the role of key cytokines secreted by WNV-infected neurons in mediating neuroinflammatory markers and neuronal death.

**Methods:**

A transformed human neuroblastoma cell line, SK-N-SH, was infected with WNV at multiplicity of infection (MOI)-1 and -5, and WNV replication kinetics and expression profile of key pro-inflammatory cytokines were analyzed by plaque assay, qRT-PCR, and ELISA. Cell death was measured in SK-N-SH cell line in the presence and absence of neutralizing antibodies against key pro-inflammatory cytokines using cell viability assay, TUNEL and flow cytometry. Further, naïve primary astrocytes were treated with UV-inactivated supernatant from mock- and WNV-infected SK-N-SH cell line and the activation of astrocytes was measured using flow cytometry and ELISA.

**Results:**

WNV-infected SK-N-SH cells induced the expression of IL-1β, -6, -8, and TNF-α in a dose- and time-dependent manner, which coincided with increase in virus-induced cell death. Treatment of cells with anti-IL-1β or -TNF-α resulted in significant reduction of the neurotoxic effects of WNV. Furthermore treatment of naïve astrocytes with UV-inactivated supernatant from WNV-infected SK-N-SH cell line increased expression of glial fibrillary acidic protein and key inflammatory cytokines.

**Conclusion:**

Our results for the first time suggest that neurons are one of the potential sources of pro-inflammatory cytokines in WNV-infected brain and these neuron-derived cytokines contribute to WNV-induced neurotoxicity. Moreover, cytokines released from neurons also mediate the activation of astrocytes. Our data define specific role(s) of WNV-induced pro-inflammatory cytokines and provide a framework for the development of anti-inflammatory drugs as much-needed therapeutic interventions to limit symptoms associated with WNVE.

## Background

West Nile virus (WNV), a mosquito-borne flavivirus that causes lethal encephalitis has emerged as a significant cause of viral encephalitis in the United States [1]. Since its introduction to North America in 1999, outbreaks of WNV fever and encephalitis have occurred in regions throughout the United States [1]. The fatality rate is approximately 10% for hospitalized encephalitic cases with increased risk in patients with compromised immune systems, older age and having underlying conditions such as diabetes mellitus [2]. Currently there are no therapeutic agents or vaccines approved for use against WNV infection in humans. Following peripheral infection, WNV replication is first thought to occur in skin Langerhans dendritic cells. These cells migrate to and seed draining lymph nodes, resulting in primary viremia [3]. By the end of the first week after infection, the virus is largely cleared from the peripheral organs, but in a subset of patients the virus enters the brain and causes a spectrum of neurological sequeale.

Major hallmarks of WNV neuropathogenesis are neuroinflammation followed by neuronal death and disruption of the blood-brain barrier (BBB) [4,5]. Activation of glial cells (microglia and astrocytes) together with neuronal death are considered as key pathogenic features of WNV neuropathogenesis [6,7]. Neuronal death in WNV infection is a complex process and involves activation of caspase3/9 dependent apoptosis via both, extrinsic as well as the intrinsic pathways [8-10]. The mechanism(s) by which WNV induces neurological sequeale are not fully understood but it is proposed that apoptotic neuronal death can be a result of both, direct virus infection or bystander injury caused by cytotoxic factors released by non-neuronal cells [11].

Though induction of neuroinflammation is an active defense reaction against insults including virus infections such as HIV, herpes simplex virus (HSV), Japanese encephalitis virus (JEV) and WNV, it is also recognized as a major contributor of neuropathogenesis [12-16]. Activated central nervous system (CNS) cells and/or infiltrating immune cells produce several proinflammatory and neurotoxic mediators including cytokines, chemokines, arachidonic acid and its metabolites [13,17]. Cytokines such as interleukin (IL)-1β and tumor necrosis factor (TNF)-α have been reported as potent inducers of neuronal injury in several neurodegenerative diseases such as cerebral ischemia, spinal cord injury, multiple sclerosis and viral infections including HIV-associated dementia (HID), JE and influenza [14,16,18,19]. The receptors of cytokines are expressed constitutively throughout the CNS, including neurons [15], thereby rendering them sensitive to these cytokines even at very low level [20].

The response of WNV infection in the brain is characterized with massive inflammatory events including production of cytokines such as IL-1β, -6 and TNF-α, and chemokines [4,11,21,22]. Studies aimed at understanding the role of innate immune response in WNV-infected brain have so far largely focused on chemokines. The role of the chemokines such as MCP-5 (or CCL12), IP-10 (or CXCL10), MIG (or CXCL9) have been established in recruitment of virus-specific T-cells and virus clearance [21,23]. However the role of pro-inflammatory cytokines such as IL-1β and TNF-α in neuropathogenesis following WNV infection remains obscure. Few studies have recently characterized the role of TNF-α in WNV infection in a mouse model, but the data remain controversial [24-26]. Although microglia and astrocytes are classically believed to serve as the predominant source of these cytokines in the CNS, neurons can highly express these cytokines in the setting of disease, including Alzheimer's disease (AD), spinal cord injury, stroke, and sciatic nerve injury [27-29]. Since neurons are the prime target for WNV replication, it seems likely that they may act as a central processor of inflammation by releasing pro-inflammatory molecules. These pro-inflammatory molecules may further activate downstream apoptotic signaling pathway(s) in neurons ultimately resulting in neuronal death and/or activate glial cells, which can further exacerbate neuroinflammation. Herein, we directly evaluate the ability of WNV-infected neurons to produce key proinflammatory cytokines and their role in mediating neuroinflammatory markers and neuronal death.

## Materials and methods

### Cells, virus and plaque assay

A transformed human neuroblastoma cell line, SK-N-SH, purchased from the American Tissue Culture Collection (ATCC, Manassas, VA) and primary human brain cortical astrocytes (HBCA) cells purchased from ACBRI (Kirkland, WA) at passage 2, were propagated as described previously [30,31]. All experiments were conducted with cells between passages 6 to 9. For infection, SK-N-SH cell line grown to 90% confluency in 6-well plates (6 × 10^5 ^cells/well) were infected with WNV (NY99) at the multiplicity of infection (MOI)-1 or -5 as described previously [30,32]. Briefly, the virus was adsorbed for 1 h at 37°C. After incubation, unadsorbed virus was removed by washing twice with PBS and cells were further incubated with fresh media. The supernatant and cells were collected at 2 h and from days 1 to 4. Production of infectious virus in the supernatant was determined by plaque assay using Vero cells as described previously [30,32].

### Quantitative real time reverse transcriptase-PCR (qRT-PCR) analysis

cDNA synthesized from RNA extracted from human neuroblastoma cell line, SK-N-SH, and HBCA cells under different conditions were used for qRT-PCR as described previously [30-33]. Primer sequences and annealing temperatures employed for amplification of pro-inflammatory cytokines are described in Table 1.

**Table 1:** Primer sequences used for qRT-PCR

Gene	Primer Sequence (5'-3')	Amplicon	
**[GenBank No.]**		**(bp)**	**Tm (°C)**

			

**IL-1β**			

**[GenBank:**NM_000576**]**			

Forward	AGCACCTTCTTTCCCTTCATC	86	56

Reverse	GGACCAGACATCACCAAGC		

			

**IL-6**			

**[GenBank:**NM_000600**]**			

Forward	CCAGGAGCCCAGCTATGAAC	84	57

Reverse	CCCAGGGAGAAGGCAACTG		

			

**IL-8**			

**[GenBank:**NM_000584**]**			

Forward	GAACTGAGAGTGATTGAGAGTGGA	91	55

Reverse	CTCTTCAAAAACTTCTCCACAACC		

			

**TNF-α**			

**[GenBank:**NM_000594**]**			

Forward	CCTGCCCCAATCCCTTTATT	81	55

Reverse	CCCTAAGCCCCCAATTCTCT		

### Treatment of WNV-infected human neuroblastoma cell line, SK-N-SH, with specific neutralizing antibodies against pro-inflammatory cytokines and cytotoxicity assay

SK-N-SH cells seeded on cover slips in 24-well plates (6 × 10^4 ^cells/well) or in 96-well plates (2 × 10^4 ^cells/well) were infected with WNV at MOI-1. After infection, the cells were replenished with either fresh media only or media containing specific neutralizing antibodies against IL-1β (Sigma), -6, -8 or TNF-α (R&D Systems). The concentration of IL and TNF-α antibodies employed in this study was 4 and 10 μg/mL, respectively. Cell viability was assessed at days 1 to 3 after infection using CellTiter 96 AQ_ueous _One Solution Cell Proliferation Assay (Promega) as described previously [30]. At day 2, cells were also fixed with 4% PFA for 10 min at room temperature (RT) for TUNEL assay.

### Treatment of naïve HBCA cells with WNV-infected UV-inactivated supernatant from human neuroblastoma cell line, SK-N-SH, and cytotoxicity assay

Supernatant derived from WNV-infected SK-N-SH cells cultured for 48 h was UV-inactivated in order to inactivate WNV and virus inactivation was confirmed by plaque assay as described previously [32]. HBCA cells grown in 24-well plates (6 × 10^4 ^cells/well) were incubated for 6 h with 500 μL of the above UV-inactivated supernatant from mock- or WNV (MOI-1) -infected SK-N-SH cells. After 6 h of treatment with UV-inactivated supernatant, HBCA cells were washed once with 1× PBS, followed by incubation at 37°C with HBCA culture media. After 24 h and 48 h, HBCA cells were harvested and supernatant was collected and stored for later use. Similarly, HBCA cells grown in 96-well plates were treated as above and cell viability assay was conducted as described previously [30].

### ELISA

The levels of IL-1β, -6, -8 and TNF-α were measured in the cell supernatant by ELISA, using the Quantakine kits (R&D Systems). The tests were conducted according to the manufacturer's instructions and the plates were analyzed using a Victor 3 microtiter reader equipped with Workout 1.5 software as described previously [31].

### TUNEL assay

Apoptosis in SK-N-SH cells treated as described above was measured using the TUNEL assay. TUNEL assay was conducted using the In Situ Cell Death Detection kit, TMR red (Roche Diagnostics, Indianapolis, IN) in accordance with the manufacturer's protocol. In brief, PFA fixed cells were permeabilized for 2 min in 0.1% Triton X-100/sodium citrate at 4°C. Cells were further incubated with 50 μL of TUNEL reaction mixture containing TdT (Terminal Deoxynucleotidyltransferase) in a humidified chamber at 37°C, washed twice with 1× PBS and then counterstained with bisbenzidine (1 ng/mL) before mounting onto a slide with Vectashield mounting medium (Vector Laboratories, Burlingame, CA). For negative control, cells were processed using a reaction mixture that did not contain TdT. For positive control, cells were incubated with *DNase I *(3 U/mL) for 10 min to induce DNA strand breaks. Apoptosis was detected by fluorescence microscopy on a Zeiss Confocal Pascal equipped with a Zeiss Axiovert 200 microscope, equipped with appropriate fluorescence filters and objectives. The TUNEL-positive cells for each group was obtained by counting total 2,500 to 3,500 cells from nine different fields for each coverslip from three independent experiments.

### Flow Cytometry

Flow cytometry was conducted using the In Situ Cell Death Detection kit, Fluorescein (Roche Diagnostics, Indianapolis, IN) to detect TUNEL-positive SK-N-SH cells that had been treated as described above. SK-N-SH cells were trypsinized, washed twice with cold PBS before fixing in 4% PFA solution. Cells were further incubated with 50 μL of TUNEL reaction mixture containing TdT (Terminal Deoxynucleotidyltransferase) in a humidified chamber at 37°C. Cells were washed and resuspended in 200 μL of PBS, and flow cytometry was conducted using the FACSAria (BD Biosciences).

In another set of experiment, HBCA cells treated with UV-inactivated supernatant from SK-N-SH cells as described previously were trypsinized at 48 h and washed twice with cold PBS before fixing in 4% PFA solution. Cells were then incubated first with monoclonal human anti-glial fibrillary acidic protein (GFAP) antibody (1:1000, DakoCytomation) at 4°C for 1 h and then with Alexa Fluor 546 conjugated goat anti-rabbit secondary antibody (1:2000, Invitrogen) for 20 min at 4°C in dark. Cells were washed and resuspended in 200 μL of PBS, and flow cytometry was conducted as described above.

### Statistical analysis

Data are reported as mean ± standard deviation (SD) of at least three independent experiments performed in duplicate. Unpaired student t-test using GraphPad Prism 5.0 (GraphPad software) was used to calculate p values. Differences of P < 0.05 were considered significant.

## Results

### WNV can infect and replicate in human neuroblastoma cell line, SK-N-SH

**Figure 1:**
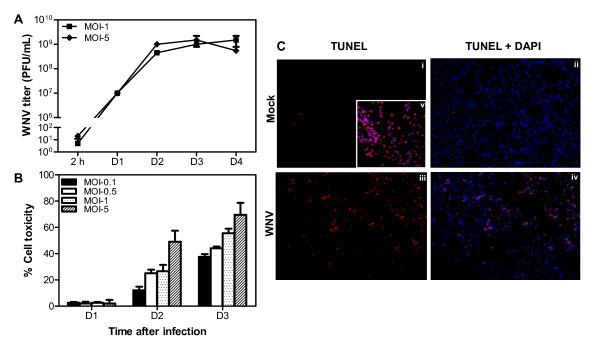
**WNV can infect and induce apoptosis in human neuroblastoma cell line, SK-N-SH**. **(A) **WNV titers in culture supernatant from SK-N-SH cell line collected at 2 h and from days 1 to 4 after infection were determined by plaque assay using Vero cells. Viral titers are expressed as plaque forming units (PFU)/mL of supernatant. Data are expressed as mean ± SD for two independent experiments conducted in duplicate. **(B) **Cell toxicity of SK-N-SH cells from days 1 to 3 after WNV infection was assessed by cell proliferation assay and percentage cell toxicity was calculated by comparing to mock-infected cells at corresponding time points. Data are expressed as mean ± SD for three independent experiments conducted in triplicate. **(C) **Mock (i and ii), and WNV (MOI-1)-infected SK-N-SH cells (iii and iv) were fixed at day 2 after infection and TUNEL assay was conducted (red; i and iii). Cells were counterstained with DAPI to label nucleus. TUNEL plus DAPI images (ii and iv) indicate that number of cells in each field were similar. *DNase I*-treated cells were used as positive control (v). The images depict representative results of three independent experiments.

We examined the susceptibility of SK-N-SH cells to WNV infection and characterized the kinetics of virus replication. Replication kinetics was analyzed by measuring WNV titer in the culture supernatants of infected cells collected at 2 h and from days 1 to 4 after infection. Plaque assay data demonstrated a robust increase in virion production from days 1 to 4 after infection with WNV at both, MOI-1 and -5 (Figure 1A). A seven-log_10 _PFU/mL increase in virus titer was observed at day 1 after infection in MOI-1 and -5 infected cells. At day 3, virus titer reached its peak of nine-log_10 _PFU/mL in MOI-5 infected cells, which declined slightly at day 4. In MOI-1 infected cells, a sharp increase in the virus titer (two-log_10 _PFU/mL) was observed on days 2 and 3 after infection, which remained elevated till day 4 after infection. The kinetics of virus replication was similar to previously observed WNV infection of primary neurons and cell lines such as LAN-2 [11,22,34].

### WNV induces apoptosis of human neuroblastoma cell line, SK-N-SH

WNV is known to induce neuronal cell death. Therefore, we examined the effect of WNV infection on cell viability of SK-N-SH cells. Cytotoxicity of WNV-infected SK-N-SH cells at different MOIs demonstrated an increase in the cell death in a dose- and time-dependent manner. As illustrated in Figure 1B, at day 1 there was no change in the cell viability of WNV-infected cells when compared to the control cells. However, at day 2 after infection with WNV at MOI-0.1, -0.5, -1, and -5, the cell toxicity increased to 14%, 25%, 26%, and 49% respectively, which continued to increase in a dose-dependent manner till day 3 after infection. This drastic increase in the cell death correlates well with the sharp increase in virus titers at the same time points (Figure 1A), further confirming the effect of WNV in inducing cell death.

To further determine that WNV-induced cell death is by apoptosis, we conducted TUNEL assay to detect DNA fragmentation, a hallmark of apoptosis. As shown in Figure 1C, very few TUNEL-positive cells were detected in mock-infected cells whereas at day 2 after WNV infection approximately 25% cells were TUNEL positive. Consistent with previously published studies, our data provides further support that apoptosis is the main mechanism by which WNV triggers neurotoxicity [9,11,34].

### WNV induces expression of multiple pro-inflammatory cytokines in human neuroblastome cell line, SK-N-SH

**Figure 2:**
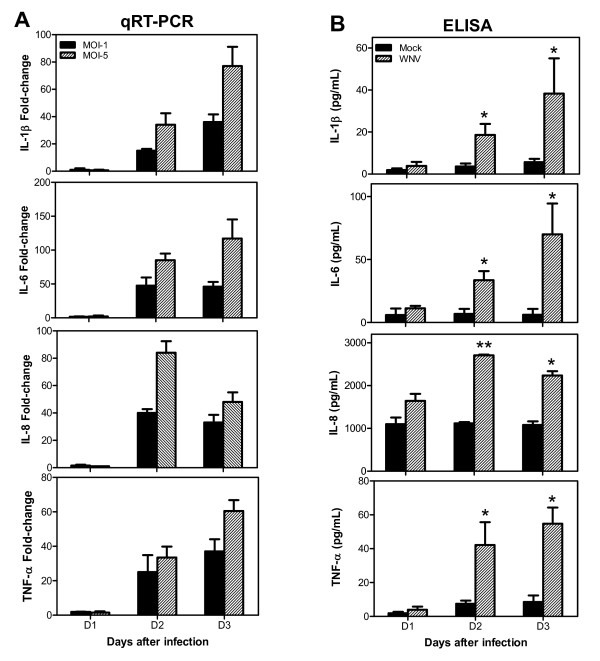
**WNV differentially modulates the expressions of pro-inflammatory cytokines in human neuroblastoma cell line, SK-N-SH**. **(A) **cDNA templates from mock- and WNV-infected SK-N-SH cells from days 1 to 3 after infection were used to determine the fold-change of IL-1β, -6, -8, and TNF-α by qRT-PCR. Changes in the levels of pro-inflammatory cytokines were first normalized to the GAPDH gene and the fold-change in infected cells as compared to corresponding controls was calculated. Data represents mean ± SD of five independent experiments conducted in duplicate. **(B) **Levels of IL-1β, -6, -8, and TNF-α in culture supernatants were determined by ELISA. WNV (MOI-1) infection significantly increased the production of pro-inflammatory cytokines. The data expressed are the mean concentration (pg/ml) ± SD of the amount of IL-1β, -6, -8 or TNF-α, secreted in the supernatant and is representative of three independent experiments. *p < 0.05. **p < 0.001.

Pro-inflammatory cytokines such as IL-1β and TNF-α play an important role in mediating neuronal death and neuroinflammation in various neurodegenerative diseases [14,17-19,35]. Therefore, we investigated the effect of WNV infection on the expression of key pro-inflammatory cytokines such as IL-1β, -6, -8, -18 and TNF-α in human neuroblastoma cell line SK-N-SH at both mRNA and protein levels. At day 1 after infection no significant increase was observed in the mRNA expression of any pro-inflammatory cytokines in both MOI-1 and -5 infected cells (Figure 2A). While IL-18 expression did not change at any time point (data not shown), a robust up-regulation was detected in the expressions of IL-1β, -6, -8, and TNF-α at days 2 and 3 after infection with both MOI-1 and -5 of WNV, which coincided with increase in cell toxicity following WNV infection (Figure 1B). Moreover, in accordance with the cell toxicity data, induction in these cytokine expressions was more in MOI-5 as compared to MOI-1 infected cells. As depicted in Figure 2A, mRNA expressions of IL-1β, -6 and TNF-α increased gradually from days 2 to 3 in both MOI-1- and -5-infected cells. Maximum increase in the expression of IL-1β, -6 and TNF-α mRNA was in the range of 60- to 120-fold, at day 3 in MOI-5 infected cells. Maximum increase in the expression of IL-8, 84-fold, was detected at day 2 in MOI-5 infected cells, which decreased to 48-fold at day 3, whereas IL-8 expression remained approximately same at both days 2 and 3 in MOI-1-infected cells.

As these pro-inflammatory cytokines are secreted proteins, their release in the culture media of mock- and WNV (MOI-1) -infected SK-N-SH cells was detected using ELISA. In controls, basal levels of IL-1β, -6 and TNF-α in cell culture media were very low. On the other hand, significant amounts of soluble IL-1β, -6 and TNF-α were detected in supernatant from infected cells at day 2 and 3 after infection (p < 0.05, Figure 2B). In accordance with mRNA data, release of these cytokines was increased at day 2 and peaked at day 3 after infection. In contrast, basal level of IL-8 was relatively high, but it also increased substantially after WNV infection, at days 2 and 3 after infection (p < 0.001, Figure 2B).

### Neutralization of IL-1β and TNF-α protects human neuroblastoma cell line, SK-N-SH, cell death

**Figure 3:**
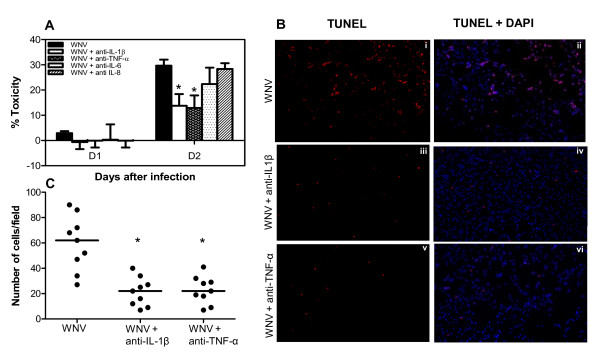
**Neutralization of IL-1β and TNF-α protects cell death of human neuroblastoma cell line, SK-N-SH**. **(A) **WNV (MOI-1)- infected SK-N-SH cells were treated with neutralizing antibodies against IL-1β-, -6-, -8-, or TNF-α and cell toxicity was assessed by cell proliferation assay. Percentage cell toxicity was calculated by comparing to control cells. While neutralization of IL-8 and IL-6 did not result in significant reduction in WNV-induced cell death, it was significantly attenuated in the presence of anti-IL1β or -TNF-α at day 2 after infection. Data are expressed as mean ± SD from three independent experiments conducted in triplicates. **(B) **WNV (MOI-1)-infected SK-N-SH cells were treated with anti-IL-1β or -TNF-α and assessed for apoptosis at day 2 after infection. Abundant TUNEL-positive cells (red, i) co-localized with DAPI (blue; ii) were observed in WNV-infected cells. In contrast, apoptosis induced by WNV was significantly attenuated in the presence of anti-IL-1β (iii) and anti-TNF-α (v). The images depicted are representative results of three independent experiments. **(C) **Quantitative representation of TUNEL-positive cells in each group from three independent cover slips from three independent experiments. Quantitative analysis indicated that neutralization of IL-1β and TNF-α reduced the number of TUNEL positive cells to less than half compared to WNV-infected cells. *p < 0.05.

To characterize the potential relationship between WNV-induced neuronal death and increased expression of IL-1β, -6, -8, and TNF-α, SK-N-SH cells were infected with WNV at MOI-1, in presence or absence of IL-1β, -6-, -8-, or TNF-α-neutralizing antibodies. Incubation of cells with IL-1β-neutralizing antibody resulted in significant (p < 0.05) reduction of the neurotoxic effects of WNV infection. The cell toxicity decreased by 50% at day 2 after infection in IL-1β-neutralizing antibody treated WNV-infected SK-N-SH cells when compared to WNV-infected SK-N-SH cells. Neutralization of TNF-α also significantly (p < 0.05) rescued WNV-induced cell death at day 2 after infection ( < 50% when compared to WNV-infected cells). On the other hand, protection of cells in the presence of anti-IL-6 and -8 for virus-induced cell death was not significant (Figure 3A).

**Figure 4:**
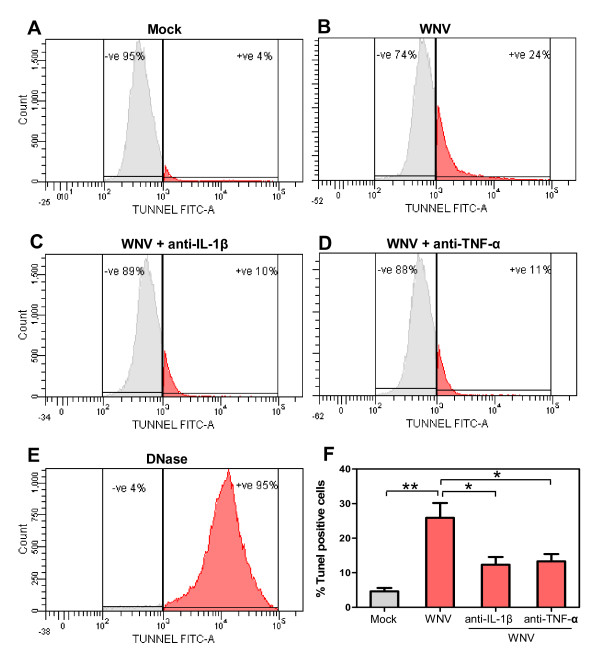
**Protection of human neuroblastoma cell line, SK-N-SH, toxicity as assayed by FACS analysis**. FACS analysis of TUNEL-positive cells in **(A) **mock and **(B) **WNV (MOI-1)-infected SK-N-SH cells treated with **(C) **anti-IL-1β or **(D) **anti-TNF-α at day 2 after infection. **(E) ***DNase I*-treated cells were used as positive control. The images depicted in panels A to E are representative data of three independent experiments. **(F) **TUNEL-positive cells reduced significantly in the presence of neutralizing antibodies against IL-1β and TNF-α. Data in panel F are expressed as mean ± SD for three independent experiments conducted in duplicate. **p < 0.001 as compared to mock, *p < 0.05 compared to corresponding infected cells.

The specificity of the neuroprotective role elicited by IL-1β and TNF-α neutralizing antibodies was further confirmed by TUNEL assay. As depicted in Figure 3B, TUNEL-positive cells were abundant in WNV-infected cells at day 2 after infection, which reduced significantly (p <0.05) in the presence of anti-IL-1β or -TNF-α. As demonstrated in Figure 3C, the number of TUNEL-positive cells in WNV-infected SK-N-SH cells treated with anti-IL-1β or TNF-α at day 2 were significantly (p < 0.05) lower, 26 and 28 cells per field, respectively, as compared to WNV-infected cells, 68 cells per field. Consistent with the cell toxicity data, the results of TUNEL assay confirmed that the neutralization of IL-1β and TNF-α protected SK-N-SH cells against WNV-induced apoptosis. The specificity of neutralizing antibodies in protecting cell death was further confirmed using flow cytometry. Flow cytometry data demonstrated that WNV-induced SK-N-SH cells apoptosis (25.8%) was significantly suppressed in the presence of neutralizing antibodies against IL-1β (12.3%) and TNF-α (13.5%) (p < 0.05, Figure 4) at day 2 after infection.

### Pro-inflammatory mediators released from WNV-infected human neuroblastoma cell line, SK-N-SH, activate naïve HBCA cells

Human astrocytes produce a wide variety of chemokines and cytokines upon exposure to pro-inflammatory stimuli. Activation of astrocytes is also one of the major hallmarks of WNV infection [11,22,34]. Therefore, we next investigated the contribution of the pro-inflammatory cytokines released from WNV-infected SK-N-SH cells in mediating neuroinflammatory markers, as assessed by activation of astrocytes and release of various pro-inflammatory cytokines.

**Figure 5:**
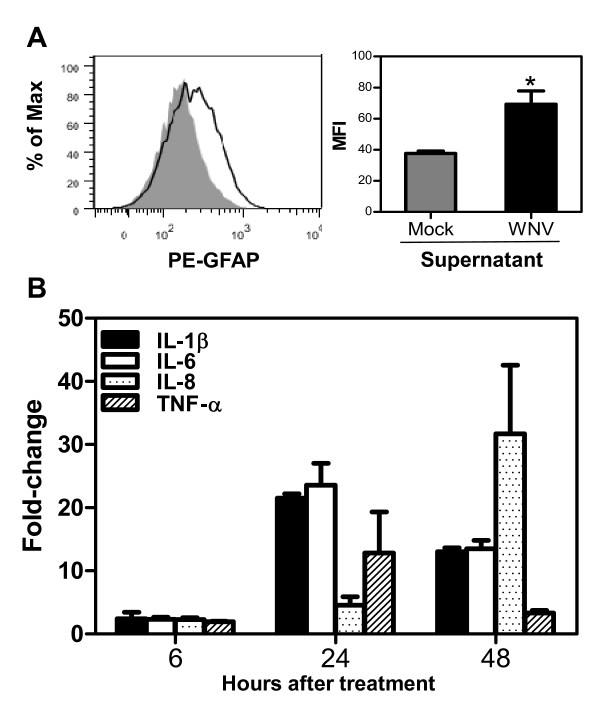
**UV-inactivated supernatant from WNV-infected human neuroblastoma cell line, SK-N-SH, activates astrocytes and induces expression of pro-inflammatory cytokines**. **(A) **FACS analysis of GFAP expression in naïve HBCA cells treated with UV-inactivated supernatant derived from mock- and WNV (MOI-1)-infected SK-N-SH cells at 48 h after treatment is shown as overlapped histograms with the mean fluorescence intensity (MFI) in arbitrary units at the right. The MFI of GFAP increased significantly in HBCA cells treated with UV-inactivated supernatant from infected SK-N-SH cells (*p < 0.05). Data are representative of three independent experiments. **(B) **cDNA templates synthesized from RNA extracted from HBCA cells at 6, 24 and 48 h after treatment with UV-inactivated supernatant from SK-N-SH cells were used to determine the fold-change of IL-1β, -6, -8, and TNF-α by qRT-PCR. Changes in the levels of pro-inflammatory cytokines were first normalized to the GAPDH gene and then the fold-change in infected supernatant treated cells as compared to corresponding controls was calculated. Data represents mean ± SD of five independent experiments conducted in duplicate.

**Figure 6:**
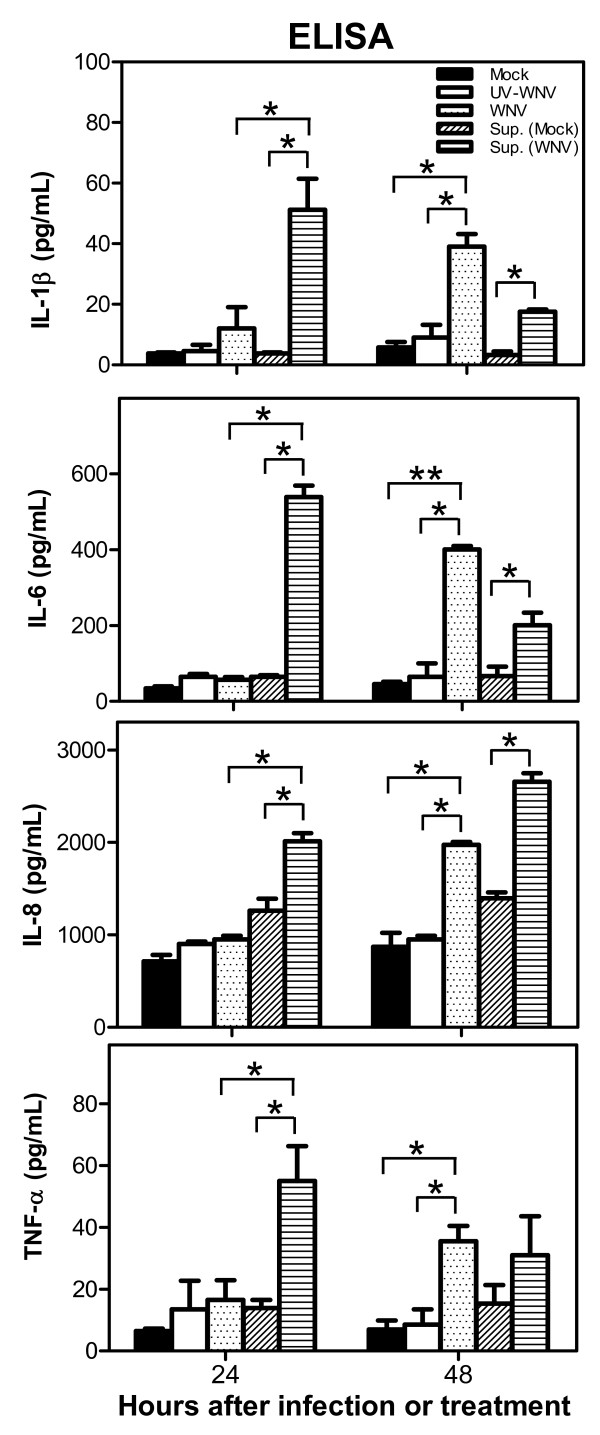
**UV-inactivated supernatant from WNV-infected human neuroblastoma cell line, SK-N-SH, induce the secretion of pro-inflammatory cytokines from astrocytes**. Naïve HBCA cells were either mock-treated or infected with UV-inactivated WNV or WNV at MOI-1 or treated with UV-inactivated supernatant derived from mock- and WNV (MOI-1)-infected SK-N-SH cells, and levels of IL-1β, -6, -8, and TNF-α in treated or infected HBCA culture supernatants were determined by ELISA. Supernatant derived from UV-inactivated mock- and WNV-infected SK-N-SH cells significantly increased the production of pro-inflammatory cytokines within 24 h after treatment. While WNV infection *per se *increased the production of these cytokines only after 48 h. Furthermore, infection of astrocytes with only UV-inactivated WNV did not induce the expression of aforementioned cytokines at any time point. The data expressed are the mean concentration (pg/ml) ± SD of the amount of IL-1β, -6, -8 or TNF-α, secreted in the supernatant and is representative of three independent experiments. *p < 0.05. **p < 0.001.

As depicted in Figure 5A, the intensity of GFAP fluorescence increased significantly (p < 0.05) in HBCA cells treated with UV-inactivated infected SK-N-SH cells supernatant (68.1%) as compared to those treated with UV-inactivated mock-infected SK-N-SH cells supernatant (46.9%) at 48 h after treatment. Figure 5B depicts sharp increase in the production of IL-1β, -8, -6, and TNF-α within 24 h of treatment of naïve HBCA cells with UV-inactivated supernatant from WNV-infected SK-N-SH cells when normalized with those treated with UV-inactivated supernatant from mock-infected SK-N-SH cells. We then analyzed the amount of these cytokines released in naïve HBCA cells treated with UV-inactivated supernatant derived from SK-N-SH cells and compared it to the kinetics of cytokine production by HBCA cells directly infected with WNV (MOI-1). In accordance with the mRNA data, the release of IL-1β, -6, -8 and TNF-α from HBCA cells treated with UV-inactivated supernatant derived from WNV-infected SK-N-SH cells was comparatively much higher than those treated with mock-infected SK-N-SH cells supernatant after 24 and 48 h as measured by ELISA (p < 0.05, Figure 6). It was interesting to note that all cytokines produced by WNV-infected HBCA cells were not significantly high at 24 hr after infection and a sharp increase occurred only at 48 h after infection, (Figure 6) which coincided with the peak in virus replication [31]. Furthermore, infection of astrocytes with only UV-inactivated WNV (MOI-1) did not induce the expression of aforementioned cytokines at any time point (Figure 6) thus ruling out the contribution of UV-inactivated WNV in inducing this effect on HBCA cells. These data collectively demonstrate that the supernatant derived from WNV-infected SK-N-SH cell line is capable of activating HBCA cells.

## Discussion

WNV-infection of brain leads to induction of several cytokines and chemokines, which promote WNV-CNS invasion and trigger neuroinflammation [5,11,23]. As a consequence, leukocytes are recruited to the CNS, which are critical for virus clearance [36,37]. However what remains unclear is the extent to which this inflammation contributes to disease pathology and poor prognosis [38,39]. The relative contribution of resident CNS cells, specifically neurons as a mediator of this inflammation is unknown. Here we demonstrate that (i) WNV-infected SK-N-SH cells are one of the potential sources of inflammatory cytokines, (ii) WNV-induced pro-inflammatory cytokines contribute to SK-N-SH cell death and glial cells activation, and (iii) WNV-induced SK-N-SH cell death can be protected in the presence of neutralizing antibodies against IL-1β and TNF-α.

### Neurons as one of the potential sources of pro-inflammatory cytokines in WNV-infected brain

Increased production of cytokines in the brain is a common event observed during infection with neurotropic viruses such as herpesviruses, JC virus, retroviruses (HIV and HTLV-1), poliovirus, rabies virus and arboviruses (JE, St. Louis encephalitis) [12-14,16,40]. Inflammation in the brain is usually characterized by infiltration of immune cells, which along with glial cells are key contributors of these cytokines [13,41,42]. We and others have recently demonstrated *in vitro *increased production of cytokines and matrix metalloproteinases (MMPs) by WNV-infected astrocytes and microglia [11,22,31]. Since neurons are the prime target of WNV infection, we examined the ability of WNV-infected human neuroblatostoma cell line, SK-N-SH, in producing key cytokines. In this report for the first time we demonstrate that neurons also respond to WNV infection by up-regulating cytokines production. The sharp increase of key pro-inflammatory cytokines such as IL-1β, -6, -8 and TNF-α at day 2 after infection as determined by qRT-PCR and ELISA is significant and coincides with peak virus replication in SK-N-SH cells suggesting that active virus replication in these cells is a main determinant of cytokines up-regulation (Figure 2). Neurons secrete several cytokines under various brain insults [43]. In the normal intact CNS, neurons are the only cell type known to produce low levels of TNF-α [44,45]. IL-6 mRNA has been described within hippocampal and cerebellar neurons of the adult mammalian brain [46,47]. Treatment of neurons with S100B induces the expression of IL-1β and -6 [41,48]. Neurons highly express cytokines such as IL-6, -1β, and TNF-α in neurodegenerative diseases, including AD, spinal cord injury, stroke, and sciatic nerve injury [27-29,49]. In diseases like AD and JE, increased cytokine production by dying neurons is the main determinant of cytotoxicity [41,50,51]. It is important to note that in HIV, another neurotropic virus, which does not infect neurons, the main source of cytokine production are glial cells [14,16,52]. However unlike HIV, WNV infection of neurons is robust, therefore based on our data, it seems likely that they may be one of the main sources of cytokines in WNV-associated neuroinflammation. Though, our data does not rule out the possibility of cytokine production by glial cells and infiltrating immune cells in WNV-infected brain. Neurons have not been implicated as a source of IL-18 in various brain insults, therefore, no change in the expression of IL-18 (data not shown) is not surprising. Moreover, literature suggests glial cells as the main source of IL-18 upon various stimuli [53], including infection with JEV [16] and WNV (Verma et. al., unpublished data).

### Neurons-derived cytokines contribute to neuronal death by apoptosis

Cytokines and their respective receptors/ligands are crucial components comprising communication network in brain and immune system [18,54]. Considerable evidence indicates that when over produced, these cytokines mediate diverse range of neurodegenerative functions including disruption of the BBB, chemoattraction of peripheral immune cells and neuronal damage [35,41]. Pro-inflammatory cytokines such as IL-1β and TNF-α have been proposed as potent mediators of neuronal death in several neurodegenerative diseases like AD, traumatic brain injury, epilepsy, Parkinson's disease, stroke, HIV and JE [12-14,16]. Based on our observation of direct correlation of cytokine production from WNV-infected SK-N-SH cells with cell toxicity (Figures 1 and 2), we next investigated the effect of these cytokines by using specific neutralizing antibodies against these cytokines. Protection of cell death as well as reduction of TUNEL positive cells in the presence of anti-IL-1β and anti-TNF-α strongly suggests that WNV-induced pro-inflammatory cytokines are one of the main factors driving cell death (Figures 3 and 4). Recent studies on JEV also support the role of TNF-α, where increased expression of TNF-α receptor in neurons directly results in the initiation of death cascade via TRADD [51]. TNF-α also contributes to neuronal death in brain ischemia [55,56]. Both TNF-α and IL-1β sensitizes neurons for tat-induced apoptosis in HAD [14]. Moreover, in vivo studies in animal models of experimental autoimmune encephalomyelitis (EAE) have demonstrated that blocking of TNF-α by neutralizing antibodies, or drugs, ameliorates the disease [57-59]. Central or peripheral administration of IL-1β dramatically increases neuronal death following acute brain injury [60,61]. Studies also support that IL-1β is the key mediator of caspase-1-dependent apoptosis of neurons [62]. IL-1β has been demonstrated to cause bystander damage to JEV-infected neurons [16]. Therapeutic blockade of IL-1 receptors also conferred significant protection in a murine model of fatal alphavirus encephalomyelitis [63]. Our results demonstrating no significant change in SK-N-SH cell death in presence of neutralizing antibodies to IL-6 and -8 was not surprising, as these cytokines are not involved in inducing cytotoxicity [42]. Moreover, production of IL-6 is under the control of IL-1β and its pathogenic roles include induction of other cytokines and chemokines [18]. Our data has inherent limitation wherein we are unable to pinpoint that infected and/or uninfected SK-N-SH cells were protected from cell death.

### Cytokines secreted by WNV-infected neurons activate astrocytes

Astrocytes, the major CNS cell type provides structural support to neurons and become activated in neuroinflammatory scenarios and produce pro-inflammatory cytokines. Signals of astrocyte activation that includes pro-inflammatory cytokines and nitric oxide may result from infected or injured glial, infiltrating immune, and/or endothelial cells or from injured neurons *per se *[13,42]. WNV-induced increased production of cytokines [11,22] (Verma et. al., unpublished data) and MMPs have been demonstrated in astrocytes [31]. Activation of glial cells has been demonstrated in WNV-infected brain [6,7,64]. However, since the infection of astrocytes by WNV in vitro is limited [22,31] and has so far not been convincingly demonstrated in vivo [11,64-66], the trigger of glial cells activation might be produced by non-glial cells. Therefore we hypothesized that cytokines released from infected neurons might be one of the initiator of glial cells activation.

Our results as depicted in Figure 5A provide direct evidence that neurotoxic mediators released from infected human neuroblastoma cell line SK-N-SH can activate astrocytes as measured by significant increase in the expression of GFAP. Increased GFAP expression as a marker of astrocyte activation has been documented in various studies [67,68]. The production of inflammatory cytokines is significantly up-regulated in naïve astrocytes treated with UV-inactivated supernatant from only infected SK-N-SH cell line, not from naive SK-N-SH cells (Figures 5B and 6). Since supernatant from WNV-infected SK-N-SH cell line was UV-inactivated and UV-inactivated WNV does not induce the expression of any cytokines upon infection (Figure 6) suggests that activation of astrocytes is mediated by SK-N-SH cells-derived inflammatory mediators only and is not the effect of WNV present in UV-inactivated supernatant. In addition, when the kinetics of cytokine production by astrocytes treated with UV-inactivated infected supernatant derived from SK-N-SH cell line was compared with cytokine profile produced by direct infection of astrocytes with WNV, we observed that direct WNV infection induced cytokines only at 48 h after infection. This could be explained by the fact that WNV infection in astrocytes peaks at day 2 and coincides with cytokine production [31]. Whereas, in astrocytes treated with UV-inactivated infected supernatant from SK-N-SH cells cytokines were produced at early time point, 24 h after treatment, and fold-change of cytokine gene expressions was also significantly higher. This fold-change of cytokine gene expression decreased at 48 h after treatment which may be due to the short half-life of cytokines in UV-inactivated infected supernatant [69,70]. The cytokines and chemokines released from injured neurons have been shown to activate non-neuronal CNS resident cells [41,51,71-73]. Based on our data, we cannot pinpoint the specific neurotoxic mediator derived from infected neurons contributing to astrocytes activation. However, available literature suggests that several factors including IL-1β and TNF-α are capable of glial cells activation [41,43,74].

The subsequent consequence of astrocyte activation and downstream cascade of inflammatory cytokine production is the secondary wave of inflammation resulting in the death of neurons. Activation of glial cells is a key pathogenic feature of WNV-associated meningoencephalitis [6,7]. Neurotoxic molecules from WNV-infected astrocytes have been demonstrated to induce indirect toxicity in un-infected neurons in vitro [11]. Our recent data demonstrating the disruption of tight junctions of the BBB by MMPs released from WNV-infected astrocytes further confirms the role of astrocytes in WNV pathogenesis [31]. These data signify the importance of astrocyte activation in WNV neuropathogenesis. Similarly, a model proposed by Swarup and colleagues, also argues that neuronal apoptosis and subsequent microglia activation in JEV infection results in bystander injury of un-infected neurons [51]. Further, blocking the downstream effects of TNF-α in JEV infection resulted in abrogation of direct neuronal death as well as bystander death mediated by activated microglia [51]. Based on these reports and data reported in this study, we suggest that in WNV infection also, neurons-mediated activation of astrocytes may result in several downstream pathological events such as bystander death of neurons, increased expression of cell adhesion molecules, chemotaxis of activated as well as infected peripheral immune cells and disruption of the BBB.

## Conclusion

In summary, our data demonstrate neurons as an essential responder to innate immune response to WNV and one of the potential sources of cytokines in brain. Considerable efforts are currently directed on targeting pro-inflammatory cytokines as novel therapeutic approach for the treatment of neurodegenerative diseases [19,43]. In vitro as well as in vivo studies have established that attenuating the cytokine production in brain directly correlates to improved disease outcome in virus infection [14,16,51,52] and in neurological disease models such as EAE and multiple sclerosis (MS) [13,17]. Broad spectrum and specific anti-inflammatory drugs such as IL-1ra and TACE-inhibitors are recommended as adjunct therapy to control disease progression of various neurodegenerative diseases such as stroke, cerebral ischemia, traumatic brain injury and MS [17,19]. Currently there is no WNV vaccine for humans and once the virus enters the brain, nothing much can be done. Therefore, the significance of our studies lies in delineating specific cell types and downstream pathways associated with cytokine production in WNV-infected brain, and lay a framework for future in vivo studies using mouse model to test the ability of anti-IL and TNF drugs to improve WNV disease outcome.

## Competing interests

The authors declare that they have no competing interests.

## Authors' contributions

MK performed the majority of experiments and statistical analysis. MK, SV and VRN were involved in conceiving the study, coordinating the experiments and data analysis. MK wrote the initial version of the manuscript, which was adapted from his Master of Science thesis. All co-authors contributed to the preparation of the manuscript. VRN was responsible for editing and revising the manuscript for the final version. All authors have read and approved the final version of the manuscript.
